# Mortality from external causes in Africa and Asia: evidence from INDEPTH Health and Demographic Surveillance System Sites

**DOI:** 10.3402/gha.v7.25366

**Published:** 2014-10-29

**Authors:** P. Kim Streatfield, Wasif A. Khan, Abbas Bhuiya, Syed M.A. Hanifi, Nurul Alam, Eric Diboulo, Louis Niamba, Ali Sié, Bruno Lankoandé, Roch Millogo, Abdramane B. Soura, Bassirou Bonfoh, Siaka Kone, Eliezer K. Ngoran, Juerg Utzinger, Yemane Ashebir, Yohannes A. Melaku, Berhe Weldearegawi, Pierre Gomez, Momodou Jasseh, Daniel Azongo, Abraham Oduro, George Wak, Peter Wontuo, Mary Attaa-Pomaa, Margaret Gyapong, Alfred K. Manyeh, Shashi Kant, Puneet Misra, Sanjay K. Rai, Sanjay Juvekar, Rutuja Patil, Abdul Wahab, Siswanto Wilopo, Evasius Bauni, George Mochamah, Carolyne Ndila, Thomas N. Williams, Christine Khaggayi, Amek Nyaguara, David Obor, Frank O. Odhiambo, Alex Ezeh, Samuel Oti, Marylene Wamukoya, Menard Chihana, Amelia Crampin, Mark A. Collinson, Chodziwadziwa W. Kabudula, Ryan Wagner, Kobus Herbst, Joël Mossong, Jacques B.O. Emina, Osman A. Sankoh, Peter Byass

**Affiliations:** 1 Matlab HDSS, Bangladesh; 2 International Centre for Diarrhoeal Disease Research, Bangladesh; 3 INDEPTH Network, Accra, Ghana; 4 Bandarban HDSS, Bangladesh; 5 Chakaria HDSS, Bangladesh; 6 Centre for Equity and Health Systems, International Centre for Diarrhoeal Disease Research, Bangladesh; 7 AMK HDSS, Bangladesh; 8 Centre for Population, Urbanisation and Climate Change, International Centre for Diarrhoeal Disease Research, Bangladesh; 9 Nouna HDSS, Burkina Faso; 10 Nouna Health Research Centre, Nouna, Burkina Faso; 11 Ouagadougou HDSS, Burkina Faso; 12 Institut Supérieur des Sciences de la Population, Université de Ouagadougou, Burkina Faso; 13 Taabo HDSS, Côte d'Ivoire; 14 Centre Suisse de Recherches Scientifiques en Côte d'Ivoire, Abidjan, Côte d'Ivoire; 15 Université Félix Houphoët-Boigny, Abidjan, Côte d'Ivoire; 16 Swiss Tropical and Public Health Institute, Basel, Switzerland; 17 Kilite-Awlaelo HDSS, Ethiopia; 18 Department of Public Health, College of Health Sciences, Mekelle University, Mekelle, Ethiopia; 19 Farafenni HDSS, The Gambia; 20 Medical Research Council, The Gambia Unit, Fajara, The Gambia; 21 Navrongo HDSS, Ghana; 22 Navrongo Health Research Centre, Navrongo, Ghana; 23 Dodowa HDSS, Ghana; 24 Dodowa Health Research Centre, Dodowa, Ghana; 25 Ballabgarh HDSS, India; 26 All India Institute of Medical Sciences, New Delhi, India; 27 Vadu HDSS, India; 28 Vadu Rural Health Program, KEM Hospital Research Centre, Pune, India; 29 Purworejo HDSS, Indonesia; 30 Department of Public Health, Universitas Gadjah Mada, Yogyakarta, Indonesia; 31 Kilifi HDSS, Kenya; 32 KEMRI-Wellcome Trust Research Programme, Kilifi, Kenya; 33 Department of Medicine, Imperial College, St. Mary's Hospital, London, United Kingdom; 34 Kisumu HDSS, Kenya; 35 KEMRI/CDC Research and Public Health Collaboration and KEMRI Center for Global Health Research, Kisumu, Kenya; 36 Nairobi HDSS, Kenya; 37 African Population and Health Research Center, Nairobi, Kenya; 38 Karonga HDSS, Malawi; 39 Karonga Prevention Study, Chilumba, Malawi; 40 London School of Hygiene and Tropical Medicine, London, United Kingdom; 41 Agincourt HDSS, South Africa; 42 MRC/Wits Rural Public Health and Health Transitions Research Unit (Agincourt), School of Public Health, Faculty of Health Sciences, University of the Witwatersrand, Johannesburg, South Africa; 43 Umeå Centre for Global Health Research, Umeå University, Umeå, Sweden; 44 Africa Centre for Health and Population Studies, University of KwaZulu-Natal, Somkhele, KwaZulu-Natal, South Africa; 45 National Health Laboratory, Surveillance & Epidemiology of Infectious Diseases, Dudelange, Luxembourg; 46 School of Public Health, Faculty of Health Sciences, University of the Witwatersrand, Johannesburg, South Africa; 47 Hanoi Medical University, Hanoi, Vietnam; 48 WHO Collaborating Centre for Verbal Autopsy, Umeå Centre for Global Health Research, Umeå University, Umeå, Sweden

**Keywords:** external causes, accidents, suicide, assault, transport, drowning, Africa, Asia, mortality, INDEPTH Network, verbal autopsy, InterVA

## Abstract

**Background:**

Mortality from external causes, of all kinds, is an important component of overall mortality on a global basis. However, these deaths, like others in Africa and Asia, are often not counted or documented on an individual basis. Overviews of the state of external cause mortality in Africa and Asia are therefore based on uncertain information. The INDEPTH Network maintains longitudinal surveillance, including cause of death, at population sites across Africa and Asia, which offers important opportunities to document external cause mortality at the population level across a range of settings.

**Objective:**

To describe patterns of mortality from external causes at INDEPTH Network sites across Africa and Asia, according to the WHO 2012 verbal autopsy (VA) cause categories.

**Design:**

All deaths at INDEPTH sites are routinely registered and followed up with VA interviews. For this study, VA archives were transformed into the WHO 2012 VA standard format and processed using the InterVA-4 model to assign cause of death. Routine surveillance data also provide person-time denominators for mortality rates.

**Results:**

A total of 5,884 deaths due to external causes were documented over 11,828,253 person-years. Approximately one-quarter of those deaths were to children younger than 15 years. Causes of death were dominated by childhood drowning in Bangladesh, and by transport-related deaths and intentional injuries elsewhere. Detailed mortality rates are presented by cause of death, age group, and sex.

**Conclusions:**

The patterns of external cause mortality found here generally corresponded with expectations and other sources of information, but they fill some important gaps in population-based mortality data. They provide an important source of information to inform potentially preventive intervention designs.

Mortality from external causes – whether unintentional (such as transport-related, falls, drowning, fires and burns, venoms, and poisons) or intentional (suicides and assaults) – forms a worldwide phenomenon of considerable magnitude. Which cause categories dominate in particular places and which age-sex groups are most affected in particular populations vary widely. Fatalities due to external causes also present a non-trivial measurement issue, since instantaneous deaths in many settings are dealt with differently (e.g. by police and other authorities) as compared to deaths during or following medical treatment for injuries (typically in hospitals).

The Global Status Report on Road Safety 2013 (1) reports over 1 million people killed on the world's roads annually, with numbers rising in some countries. Despite technological improvements in vehicles and roads, increasing traffic density can bring increased risks, particularly to pedestrians. The World Health Organization (WHO) African Region is estimated to have the highest rate of road traffic deaths, at 0.24 per 1,000 population, with the South-East Asia Region at 0.18 per 1,000 population.

Child injuries have also been documented globally in the World Report on Child Injury Prevention (2). Globally, child injury deaths number close to one million per year, with the majority occurring in low- and middle-income countries. Leading cause categories are road traffic and drowning.

A review of data on suicide in Africa showed major gaps, making estimates of overall patterns uncertain (3). Published rates from various African countries ranged from 0.004 to 0.17 per 1,000 population. A global analysis of suicide estimated a rate of 0.06 per 1,000 in the WHO African Region and 0.16 in the WHO South-East Asia Region (4). The same source estimated rates for violence and war at 0.23 per 1,000 population in Africa and 0.08 per 1,000 in South-East Asia.

The INDEPTH Network works with Health and Demographic Surveillance Sites (HDSS) across Africa and Asia, which each follow circumscribed populations on a longitudinal basis. Core data collected include person-time at risk, together with deaths and, by means of verbal autopsy (VA), assessment of cause of death (5). This allows reporting of external cause mortality on the basis of individually documented deaths within defined populations, adding considerably to existing overall estimates, which are often based on health facility data.

Our aim in this article is to document deaths among entire populations in a dataset from 22 INDEPTH HDSSs covering Africa and Asia, looking particularly at those deaths attributable to external causes. We define external causes here to include all of the WHO 2012 VA standard chapter 12 causes, corresponding to ICD-10 codes S00 to Y98 (6). Although these 22 sites are not designed to be a representative sample, they enable comparisons to be made over widely differing situations, using standardised methods.

## Methods

The overall INDEPTH data set from which these analyses of external cause mortality are drawn is described in detail elsewhere (7). Across the 22 participating sites (8–29) , there is documentation on 111,910 deaths in 12,204,043 person-years of observation. These data are available in a public-domain data set (30), and the methods used to compile that data set are summarised in Box 1.

*Box 1*. Summary of methodology based on the detailed description in the introductory paper (7)

**Age–sex–time standardisation**

To avoid effects of differences and changes in age-sex structures of populations, mortality fractions and rates have been adjusted using the INDEPTH 2013 population standard (31). A weighting factor was calculated for each site, age group, sex, and year category in relation to the standard for the corresponding age group and sex, and incorporated into the overall data set. This is referred to in this article as *age-sex-time standardisation* in the contexts where it is used.

**Cause of death assignment**

The InterVA-4 (version 4.02) probabilistic model was used for all of the cause-of-death assignments in the overall data set (32). InterVA-4 is fully compliant with the WHO 2012 Verbal Autopsy (VA) standard and generates causes of death categorised by ICD-10 groups (33). The data reported here were collected before the WHO 2012 VA standard was available, but were transformed into the WHO2012 and InterVA-4 format to optimise cross-site standardisation in cause-of-death attribution. For a small proportion of deaths, VA interviews were not successfully completed; a few others contained inadequate information to arrive at a cause of death. InterVA-4 assigns causes of death (a maximum of three) with associated likelihoods; thus, cases for which likely causes did not total 100% were also assigned a residual indeterminate component. This served as a means of encapsulating uncertainty in cause of death at the individual level within the overall data set, as well as accounting for 100% of every death.

**Overall dataset**

The overall public-domain data set (30) thus contains between one and four records for each death, with the sum of likelihoods for each individual being unity. Each record includes a specific cause of death, its likelihood, and its age-sex-time weighting.

Deaths assigned to any of the WHO 2012 VA cause-of-death categories relating to external causes (VA12.01 to VA12.99) were extracted from the overall database, together with details of site, age group at death, and sex. Person-year denominators corresponding to the same categories were included from the corresponding surveillance data.

Of the 22 sites reported in the data set, two (FilaBavi, Vietnam; Niakhar, Senegal) reported very few deaths due to external causes, accompanied by little specific information as to cause of death. These did not provide a credible picture of mortality from external causes, and consequently the following analyses are based on data from the remaining 20 sites, relating to 5,884 deaths over 11,828,253 person-years observed. The Karonga, Malawi, site did not contribute VAs for children. Sites reported for different time periods; overall, 5.0% of the person-time observed occurred before 2000, 28.2% from 2000 to 2005, and 66.7% from 2006 to 2012. As each HDSS covers a total population, rather than a sample, uncertainty intervals are not shown.

In this context, all of these data are secondary data sets derived from primary data collected separately by each participating site. In all cases, the primary data collection was covered by site-level ethical approvals relating to ongoing health and demographic surveillance in those specific locations. No individual identity or household location data were included in the secondary data, and no specific ethical approvals were required for these pooled analyses.

## Results

**Fig. 1:**
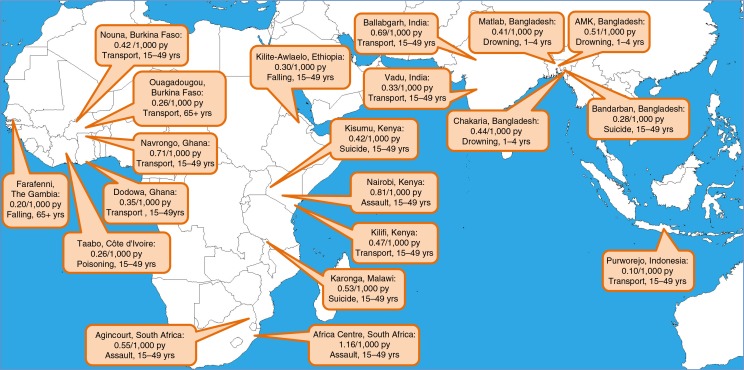
Map showing overall age-sex-time standardised mortality rates per 1,000 person-years due to external causes, also listing the specific cause category and age group accounting for the largest proportion of deaths due to external causes at each site, for 20 INDEPTH sites.

Table 1 shows the overall numbers of deaths from external causes and the exposure time for each site, by age group. Figure 1 shows a map of the 20 sites, each one marked with its age-sex-time standardised overall mortality rate for deaths due to external causes, plus a note of the specific WHO 2012 VA external cause category and age group which accounted for the largest proportion of overall deaths from external causes. Approximately one-quarter of deaths due to external causes occurred in the under-15-year age group. External cause mortality at three of the Bangladeshi sites was dominated by drownings among small children, while elsewhere leading cause categories mainly comprised transport-related deaths, suicides, and assaults.

**Table 1:** Numbers of deaths from external causes and person-years (py) of exposure, by age group, for 20 INDEPTH sites

	Infants		1–4 years		5–14 years		15–49 years		50–64 years		65+years	
	
	Deaths	py	Deaths	py	Deaths	py	Deaths	py	Deaths	py	Deaths	py
Bangladesh: Matlab	12.4	41,792	242.5	167,334	88.7	401,272	202.3	886,951	56.4	189,069	78.7	108,061
Bangladesh: Bandarban		1,242	2.0	5,770	2.0	13,626	10.5	30,173		5,891	2.0	2,705
Bangladesh: Chakaria	4.8	5,636	29.5	21,992	20.7	60,951	15.8	104,097	5.8	16,234	9.9	8,257
Bangladesh: AMK	2.4	10,558	60.9	43,236	25.5	105,701	112.1	274,129	20.2	53,184	25.4	26,927
Burkina Faso: Nouna	13.7	30,362	37.8	105,185	50.0	181,699	91.8	275,936	30.0	47,682	44.6	27,722
Burkina Faso: Ouagadougou	0.9	6,943	3.0	27,941	6.5	51,217	17.1	119,468	6.6	11,459	8.3	4,149
Côte d'Ivoire: Taabo		3,962	3.0	12,951	6.9	30,967	14.1	48,484		6,967	3.4	3,173
Ethiopia: Kilite Awlaelo		3,185	1.5	13,009	11.3	39,917	16.4	59,397	4.6	11,173	6.9	7,125
The Gambia: Farafenni	1.6	11,438	3.4	42,802	8.1	88,740	21.4	139,746	5.9	22,485	15.8	11,506
Ghana: Navrongo	19.2	30,124	52.3	116,283	119.3	296,767	314.7	534,464	140.7	128,494	226.6	70,664
Ghana: Dodowa	1.9	14,120	9.9	58,318	19.9	138,762	91.6	255,677	24.8	37,001	32.7	27,227
India: Ballabgarh	4.0	8,405	12.9	30,478	17.3	77,584	165.0	194,902	27.8	30,823	32.0	15,597
India: Vadu		4,285	0.0	16,484	2.0	33,973	49.7	128,387	11.4	15,518	15.8	7,469
Indonesia: Purworejo		2,845		14,350	2.6	44,166	16.4	136,422	6.7	27,091	3.2	21,793
Kenya: Kilifi	3.0	38,526	13.5	147,331	41.8	310,584	169.2	422,507	61.6	65,606	86.1	33,092
Kenya: Kisumu	21.3	39,887	57.6	144,451	41.6	324,153	202.2	467,691	60.5	89,105	73.5	67,080
Kenya: Nairobi	11.9	14,350	22.0	62,552	22.2	108,651	354.7	383,810	23.6	24,804	10.6	5,640
Malawi: Karonga							41.0	117,499	11.5	14,783	15.5	11,356
South Africa: Agincourt	8.4	36,811	28.3	148,961	58.3	369,285	565.5	725,431	90.4	92,519	65.3	63,187
South Africa: Africa Centre	7.3	22,468	34.4	91,367	69.8	232,962	544.8	374,099	92.3	54,852	87.7	39,160

**Fig. 2:**
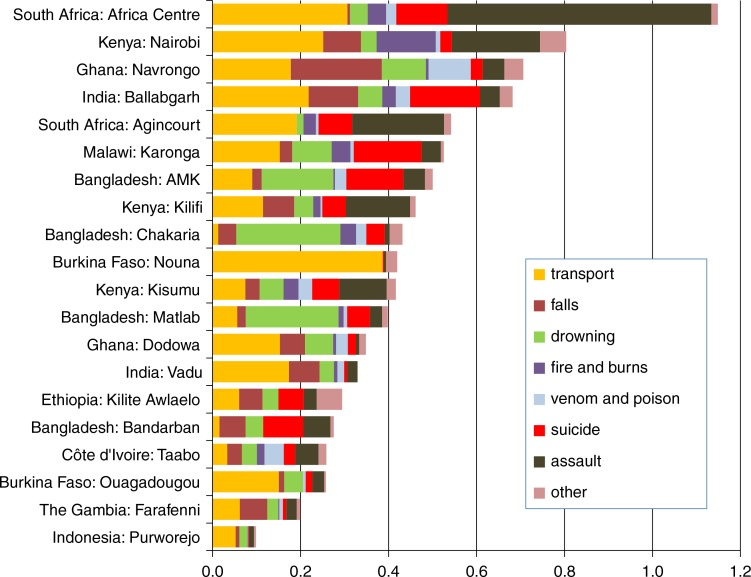
Age-sex-time standardised mortality rates per 1,000 person-years by category of external causes of death, from 20 INDEPTH sites.

Figure 2 shows the breakdown of overall external cause mortality age-sex-time standardised rates by cause category and site. At the Nouna, Burkina Faso, site, almost all external cause deaths were attributed to transport-related causes (possibly through the use of an historic VA instrument that did not contain all of the WHO 2012 VA items). Elsewhere, there were similar mixes of cause categories between sites, with some local variations.

Table 2 shows age-sex-time standardised cause-specific mortality rates by cause category, sex, and site for adults (aged 15 years and older). Men were at higher risk of transport-related death than women at every site. Suicides were most common in Bangladesh, particularly among women; in Eastern and Southern Africa, they were more common among men. Sites in Western Africa generally recorded low rates of suicide. South African men were subject to high rates of death following assault.

**Table 2:** Age-sex-time standardised mortality rates per 1,000 person-years for adults (aged 15 years and older), by sex and category of external causes of death, for 20 INDEPTH sites

	Transport		Falls		Drowning		Fire and burns		Venom and poison		Suicide		Assault		Other	
	
Site	Male	Female	Male	Female	Male	Female	Male	Female	Male	Female	Male	Female	Male	Female	Male	Female
Bangladesh: Matlab	0.12	0.02	0.03	0.01	0.04	0.02	0.01	0.01	0.00	0.00	0.05	0.10	0.04	0.03	0.02	0.01
Bangladesh: Bandarban	0.05		0.05								0.10	0.18	0.19		0.02	
Bangladesh: Chakaria	0.04		0.10	0.02	0.01	0.03		0.11	0.01		0.03	0.10	0.03	0.01	0.01	0.04
Bangladesh: AMK	0.19	0.02	0.03	0.02				0.01	0.01	0.00	0.15	0.21	0.09	0.01	0.04	
Burkina Faso: Nouna	0.62	0.38										0.01	0.01	0.01	0.04	0.03
Burkina Faso: Ouagadougou	0.42	0.05		0.01	0.01				0.02		0.03	0.02	0.09			0.01
Côte d'Ivoire: Taabo	0.06		0.05	0.04	0.06		0.03		0.09		0.06		0.15		0.02	0.04
Ethiopia: Kilite Awlaelo	0.08	0.07	0.15	0.04	0.03						0.14	0.04	0.08	0.02	0.03	0.05
The Gambia: Farafenni	0.17	0.05	0.07	0.10	0.06	0.01				0.01	0.01	0.01	0.10		0.01	0.01
Ghana: Navrongo	0.41	0.12	0.32	0.25	0.09	0.01	0.00	0.00	0.13	0.06	0.06	0.03	0.12	0.04	0.09	0.03
Ghana: Dodowa	0.39	0.11	0.08	0.08	0.11	0.02	0.01		0.07	0.02	0.05	0.02	0.01	0.01	0.04	0.01
India: Ballabgarh	0.33	0.26	0.14	0.08	0.02	0.06	0.01	0.05	0.03	0.02	0.24	0.21	0.07	0.06	0.04	0.02
India: Vadu	0.36	0.04	0.08	0.12	0.03	0.06	0.01	0.01	0.02		0.02		0.04	0.03		
Indonesia: Purworejo	0.11	0.01	0.02	0.01	0.02	0.02		0.01				0.00	0.02	0.01	0.01	0.00
Kenya: Kilifi	0.33	0.07	0.22	0.06	0.12	0.01	0.05	0.02	0.02		0.20	0.03	0.53	0.08	0.03	0.00
Kenya: Kisumu	0.21	0.04	0.05	0.05	0.10	0.02	0.01	0.02	0.04	0.02	0.19	0.05	0.34	0.05	0.04	0.01
Kenya: Nairobi	0.71	0.05	0.17	0.08	0.06		0.26	0.06	0.02	0.00	0.08	0.01	0.66	0.03	0.10	0.01
Malawi: Karonga	0.26	0.06	0.01	0.04	0.18	0.01	0.05	0.04	0.02		0.23	0.09	0.14	0.04	0.01	0.01
South Africa: Africa Centre	0.89	0.13	0.01	0.01	0.07		0.07	0.04	0.06	0.00	0.39	0.05	2.01	0.30	0.02	0.01
South Africa: Agincourt	0.38	0.12			0.01	0.00	0.02	0.04	0.01	0.00	0.11	0.12	0.48	0.17	0.02	0.02

Table 3 shows, in the same format, age-sex-time standardised cause-specific mortality rates for children. Boys generally experienced higher rates of transport-related mortality than girls, although they were lower rates than for adults. At most sites, drowning occurred at higher rates among boys.

**Table 3:** Age-sex-time standardised mortality rates per 1,000 person-years for children (aged under 15 years), by sex and category of external causes of death, for 19 INDEPTH sites

	Transport		Falls		Drowning		Fire and Burns		Venom and Poison		Suicide		Assault		Other	
	
Site	Male	Female	Male	Female	Male	Female	Male	Female	Male	Female	Male	Female	Male	Female	Male	Female
Bangladesh: Matlab	0.05	0.02	0.02	0.01	0.65	0.47	0.01	0.03	0.02	0.01	0.00	0.01	0.02	0.02	0.01	0.02
Bangladesh: Bandarban			0.25		0.12	0.11										
Bangladesh: Chakaria	0.01			0.03	0.61	0.48		0.02	0.09		0.02				0.01	0.06
Bangladesh: AMK	0.12	0.02	0.03		0.59	0.46			0.09	0.05	0.02	0.03	0.07	0.02	0.01	0.01
Burkina Faso: Nouna	0.36	0.17													0.02	0.02
Burkina Faso: Ouagadougou	0.02		0.05		0.16	0.04										
Côte d'Ivoire: Taabo	0.07			0.04	0.04	0.04		0.05		0.07	0.04		0.04			
Ethiopia: Kilite Awlaelo	0.05	0.03			0.07	0.06					0.03				0.10	0.07
The Gambia: Farafenni	0.01	0.01	0.03	0.02	0.03	0.01		0.01	0.02							0.02
Ghana: Navrongo	0.07	0.05	0.13	0.04	0.29	0.09	0.01	0.00	0.10	0.10		0.00	0.01	0.00	0.01	0.02
Ghana: Dodowa	0.03	0.04	0.03	0.02	0.09	0.06	0.01	0.01	0.02						0.01	
India: Ballabgarh	0.07	0.05	0.13	0.10	0.06	0.11	0.02	0.06	0.03	0.05	0.04					0.06
India: Vadu	0.05								0.05							0.00
Indonesia: Purworejo	0.05	0.03			0.05											
Kenya: Kilifi	0.04	0.04	0.02		0.03	0.02	0.00	0.00			0.00		0.01	0.01	0.01	0.00
Kenya: Kisumu	0.04	0.01	0.01	0.00	0.05	0.05	0.06	0.05	0.03	0.03	0.01	0.00	0.03	0.01	0.03	0.01
Kenya: Nairobi	0.16	0.03	0.01	0.03	0.10	0.02	0.13	0.10	0.02	0.01					0.07	0.09
South Africa: Africa Centre	0.12	0.12	0.01	0.00	0.06	0.06	0.02	0.04	0.02	0.02	0.02	0.01	0.06	0.05	0.00	0.02
South Africa: Agincourt	0.11	0.07			0.04	0.02	0.02	0.02		0.01	0.02		0.02	0.01	0.01	0.02

**Fig. 3:**
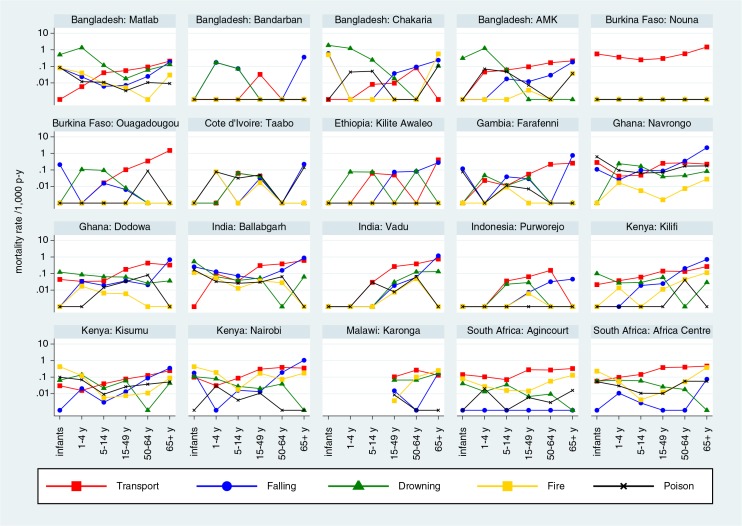
Site-specific mortality rates per 1,000 person-years by age group and category of unintentional external causes of death.

**Fig. 4:**
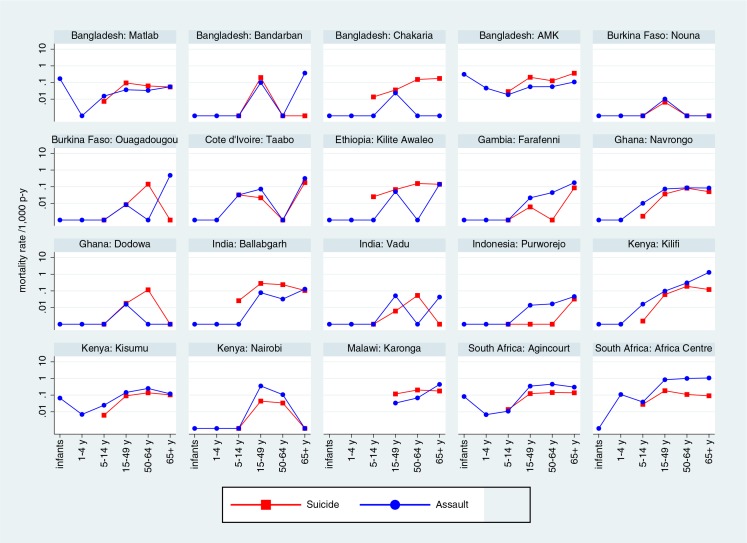
Site-specific mortality rates per 1,000 person-years by age group and category of intentional external causes of death.

Figure 3 shows site-specific mortality rates by categories of unintentional external causes and age group, with rates for all sites shown on the same logarithmic scale for ease of comparison. Figure 4 shows intentional external causes on the same basis.

## Discussion

As was clear from the overview of this cause-specific mortality data set (7), deaths due to external causes form an important component of overall mortality, and in particular account for many premature deaths, in both childhood and early adulthood. The major advantage of addressing external cause mortality from this data set, which included all deaths within circumscribed surveillance populations, is that various biases from attempting to capture injury data alone were avoided. Most obviously, it avoids the difficulties of accounting for both instantaneous fatalities and health facility deaths, which otherwise involves trying to combine diverse reporting mechanisms.

Patterns of external cause mortality revealed from these analyses were more or less consistent with the relatively few other direct measurements from Sub-Saharan Africa and South-East Asia. It is clear that geographic location, age, and sex are major determinants not only of overall external cause mortality but also of specific cause categories. In some cases, geography appeared to play a direct role, for example in the problematically high rates of child drowning in Bangladesh. At the Bandarban site, some 200 m above sea level near the Myanmar border, drowning rates were appreciably lower than in the flat river delta environments of the other three sites in Bangladesh. Similarly, in the mountainous area covered by the Kilite Awlaelo, Ethiopia, site, falling was the major cause of death. It is important to be clear that the WHO 2012 VA standard and the InterVA-4 model are designed for assigning causes of death, and not mechanisms of injury, which are consequently not discussed here.

For suicide, rates were high among Bangladeshi women, whereas in Eastern and Southern Africa rates were high among men; suicide overall was much less common in Western Africa. South Africa and Kenya showed appreciably higher rates of assault-related deaths than other countries reporting here. Countries with poorly developed road transport infrastructures, for example in Western Africa, emerged clearly with high rates of transport-related mortality.

It is sometimes assumed that external causes of death represent a relatively easy option for assigning cause of death via VA. This may be true for a proportion of deaths from external causes, for example instantaneous fatalities with no complicating factors. This assumes, however, that all deaths from external causes have reliable witnesses who can be traced for VA interviews, and who, in the case of inflicted injuries, were not the perpetrators. In this study, based on VA material derived via a variety of antecedents to the WHO 2012 VA standards, there may also have been some difficulties in extracting all the necessary data items correctly, particularly for details of injuries contained in narratives. This probably led to artefacts with road transport deaths in the Nouna, Burkina Faso, site. A few deaths at the Karonga, Malawi, site were incorrectly attributed to burns only on the basis of skin symptoms.

However, there may also be cases where not all is as it seems at first sight, and the details of these may be difficult to ascertain from VA interviews. It has been suggested that suicide rates are actually correlated with autopsy rates; in other words, methods of assigning cause of death are important, particularly when complex and sensitive issues may be involved (34). Using VA, it is very likely, for example, that fatal injuries involving a motor vehicle will be attributed to road traffic deaths, even though motor vehicles can be used as weapons of assault or instruments of suicide.

## Conclusions

The patterns of external cause mortality presented here generally conform to expectations, but at the same time they provide detail to fill in some of the gaps in knowledge about deaths arising from injuries of various kinds in Africa and Asia. Clearly, many of the specific mortality burdens identified must be considered as in principle being largely avoidable, given that they do not happen uniformly across locations and population groups. However, preventing external cause mortality poses major challenges involving social, behavioural, environmental, and regulatory considerations. Nevertheless, documenting the major targets for prevention is an important prerequisite.
